# Eliminating Trachoma in Areas with Limited Disease

**DOI:** 10.3201/eid0905.020577

**Published:** 2003-05

**Authors:** Bruce D. Gaynor, Yinghui Miao, Vicky Cevallos, Hem Jha, JSP Chaudary, Ramesh Bhatta, Susan Osaki-Holm, Elizabeth Yi, Julius Schachter, John P. Whitcher, Thomas Lietman

**Affiliations:** * University of California, San Francisco, San Francisco, California, USA; † Geta Eye Hospital, Geta, Nepal

**Keywords:** trachoma, chlamydia, azithromycin, dispatch

## Abstract

The common wisdom is that a trachoma program cannot eliminate ocular chlamydia from a community, just reduce infection to a level where there would be minimal blindness. We describe the success of multiple mass antibiotic treatments, demonstrating that complete elimination of infection may be an attainable goal in an area with modest disease.

The World Health Organization (WHO) and a number of its partners have initiated a program to eliminate blinding trachoma by the year 2020 (*1*). Many healthcare workers feel that attempting to eradicate the ocular strains of chlamydia that cause trachoma (serovars A, Ba, B, and C) would be unrealistic and perhaps even unnecessary. A more attainable goal would be to reduce clinically active trachoma to some threshold, below which scarring and blindness would never occur or at least would become so rare that trachoma would no longer be a major public health concern (*2*).

Although in common usage the terms eradication and elimination can be synonymous, in the field of public health, they are not (*3*). Both terms imply reduction of incidence to zero. However, eradication applies to the whole world, whereas elimination applies to a defined geographic area and would require further monitoring; in a sense, elimination can be viewed as a local eradication (*4*). In practice, WHO has allowed an even looser usage of the term elimination: for example, leprosy elimination is defined as a prevalence of <1 case in 10,000 population, and tuberculosis elimination is an incidence of <1 case in 100,000 persons per year (*5*,*6*). WHO is currently in the process of defining such a level for trachoma.

Defining an appropriate target for trachoma elimination is particularly difficult because infection itself is rarely monitored. Control programs rely almost exclusively on the clinical examination because the most sensitive chlamydial tests are expensive and not widely available in trachoma-endemic areas. The clinical examination is certainly a reasonable tool to assess whether ocular chlamydia is hyperendemic in a community. However, the examination may not be an accurate indicator of infection when disease prevalence is low, as is often seen after treatment (*7*–*11*). The follicles so characteristic of clinically active trachoma may linger even when chlamydia is no longer detectable by using the most sensitive laboratory techniques (*7*,*12*). The few studies that have tracked ocular chlamydial infection using DNA amplification tests suggest that a single mass antibiotic distribution is very effective, much more successful than a clinical survey would indicate (*9*,*10*).

Could ocular chlamydia be eliminated with multiple treatments? A mathematical model has shown that periodic treatments could theoretically eliminate infection even without a perfect antibiotic or perfect coverage of the population (*13*). This same model predicts that annual treatment in areas with moderate amounts of trachoma should progressively reduce the prevalence of ocular chlamydia in a community. To date, however, no reports of the efficacy of multiple annual treatments on infection have been published.

## The Study

We monitored trachoma prevalence in a village in Western Nepal for 3 years, using both clinical grading system and nucleic acid amplification tests. Three annual azithromycin (20 mg/kg) treatments were distributed to all children ages 1–10 years in the village (Figure). All children were examined biannually, and the conjunctivae of a stratified random sample of children were swabbed and later tested for *Chlamydia trachomatis* DNA. At the final visit, 6 months after the last treatment, every child was examined, and their conjunctivae were swabbed. Before the first treatment, 39% had active infection determined by the clinical examination, and an estimated 26% (95% confidence interval [CI] 16% to 35%) were infected with chlamydia. At the final, May 2001 visit, 7 (4%) of 187 pediatric cases were clinically active. Only 1 child of the 187 (0.5%) had evidence of chlamydia by polymerase chain reaction.

**Figure:**
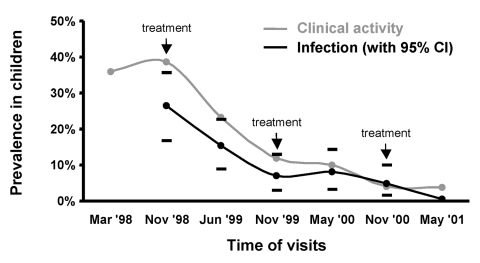
The prevalence of clinically active trachoma (gray curve) and ocular chlamydial infection, as determined by DNA amplification tests (black curve, with 95% confidence intervals due to stratified sampling) in children 1–10 years of age in a village in Western Nepal over time. All children were examined at each visit, so no sampling confidence interval is indicated. Likewise, conjunctivae of all children were swabbed for evidence of infection at the May 2001 visit.

## Conclusions

This study suggests that local elimination of the ocular chlamydia that causes trachoma may be possible in a village with moderate baseline disease. After three annual treatments, only one infected child could be identified. Children are by far the most likely to harbor ocular chlamydia, and mathematical models imply that they will be the most difficult group to clear from infection (*13*,*14*). In fact, 1 year after mass azithromycin treatment in a village in Egypt, more infection was identified in children 1–5 years old than in the rest of the community combined (*9*,*15*). Thus, the nearly complete absence of infection in children after three treatments implies that elimination is a possibility. Whether success in this village was due solely to our treatment program or due in part to a secular trend in the area, the results are encouraging.

Is elimination of ocular chlamydia necessary? It may not be for at least three reasons. First, repeat infections are almost certainly required to cause severe conjunctival scarring; occasional sporadic infections probably do not lead to blindness. Second, some investigators hope that if ocular chlamydia is reduced to a low enough level, the disease will have difficulty repopulating the community (population biologists call such a prevalence threshold an Allee effect [*16*]). While we see no reason for such a phenomenon a priori, if present, it would certainly establish a threshold target. Finally, bacterial, viral, and allergic conjunctivitides can occasionally mimic ocular chlamydia, so eradication of “clinically active” trachoma will never be possible.

Trachoma programs have already distributed more than 1 million doses of oral azithromycin, and some villages have received three annual treatments. How will we know when to stop? Now is the time to discuss the most appropriate target for trachoma programs and the most appropriate definition for trachoma elimination. The common wisdom is that complete local elimination of ocular chlamydia to zero in a defined geographic area is an unattainable goal, and that programs should settle for reducing the prevalence of ocular chlamydia to a level where little if any subsequent blindness would exist. These results from Nepal imply that the strict definition of elimination of ocular chlaymdia in children may be an attainable goal, at least in areas with modest to moderate disease. Whether or not elimination is necessary is a separate question.
